# Multi‐host lifestyle in plant‐beneficial bacteria: an evolutionary advantage for survival and dispersal?

**DOI:** 10.1111/1462-2920.16002

**Published:** 2022-04-12

**Authors:** Miguel A. Matilla, Amalia Roca

**Affiliations:** ^1^ Department of Environmental Protection Estación Experimental del Zaidín, Consejo Superior de Investigaciones Científicas Granada Spain; ^2^ Department of Microbiology, Facultad de Farmacia, Campus Universitario de Cartuja Universidad de Granada Granada 18071 Spain

Plants harbour a wide diversity of microorganisms that efficiently colonize different internal and external plant organs and compartments, including the phyllosphere (above‐ground plant surface), spermosphere (seeds and area surrounding seeds), endosphere (internal tissues) and rhizosphere (roots and soil in the vicinity of plant roots), establishing complex and dynamic interactions with the host plants (Trivedi *et al*., 2020). The plant microbiome plays major roles in the nutrition, growth and resistance against biotic and abiotic threats (Trivedi *et al*., 2020; Bakker and Berendsen, 2022; Yuan *et al*., 2022) and there is complex communication between microorganisms and their plant hosts (Berlanga‐Clavero *et al*., 2020; Rico‐Jiménez *et al*., 2022). Indeed, the secretion of a great variety of plant compounds directs the assembly of plant‐associated microbial communities and it has been proposed that plants produce a range of chemical signals to selectively recruit specific microorganisms in order to assemble protective microbiomes that enable them to cope with the imposed biotic and abiotic stresses (Rizaludin *et al*., 2021; Rolli *et al*., 2021; Trivedi *et al*., 2022). As a consequence of this selective pressure exerted by the plants, the microbial composition of the rhizosphere and the non‐rooted bulk soil differ – with the rhizosphere having a larger microbial abundance but lower diversity (Berlanga‐Clavero *et al*., 2020; Sokol *et al*., 2022).

Additionally, plants interact with higher organisms, including insects. The microbiome of a host insect is also critical for overcoming multiple stresses and plays key roles in insect metabolism, growth and defence (Engel *et al*., 2016; Liu *et al*., 2019). For example, specific phytotoxin‐degrading microbes present in the guts of insect herbivores confer resistance to these toxins, therefore allowing these herbivores to diversify and broaden the number of host plants (Itoh *et al*., 2018). Notably, an increasing amount of experimental data suggests a link between the microbiota of soils, plants and plant‐associated insects (Liu *et al*., 2019), which further reflects the complexity of interactions that occur in plant environments. In fact, a recent study revealed that monarch butterflies that feed on milkweed plants (*Asclepias* spp.) share a high percentage of bacterial taxa with the rhizosphere of the host plants (Hansen and Enders, 2022).

Currently, research on the transfer of microbial communities from plants to insects (and *vice versa*) remains almost exclusively restricted to plant pathogens and insect pests. For example, a hot topic in the recent years has been the ecology and virulence of *Xylella fastidiosa* – an important re‐emerging phytopathogen which is obligatorily transmitted to the host plant by xylem sap‐sucking insects (Sicard *et al*., 2018). However, research involving interactions between insects and beneficial plant‐associated bacteria remains poorly investigated. Notably, in a recent review published in *Environmental Microbiology*, Pronk and co‐workers (2022) critically analyse different examples of plant‐beneficial bacteria which successfully colonize co‐occurring insects. Contrary to what occurs with their plant hosts, plant‐beneficial bacteria can establish commensal, mutualistic or pathogenic interactions with their insect hosts. One of these examples involves biocontrol rhizosphere bacteria of the *Pseudomonas protegens* species, which can switch from a root‐ to an insect‐associated lifestyle (and *vice versa*). *Pseudomonas protegens* isolates were shown to invade and kill insects by producing multiple bioactive molecules and exoenzymes. It was also found that *P*. *protegens* strains can multiply at high levels in the insect corpse, as a prior step to rhizosphere re‐colonization. This host alternation would potentially allow beneficial plant‐associated bacteria to successfully disperse to other plants over long distances (Pronk *et al*., 2022). The dual lifestyle of plant‐ and insect‐associated bacteria was also analysed and discussed by Pronk *et al*. (2022) for strains belonging to the *Bacillus*, *Burkholderia*, *Photorhabdus* and *Streptomyces* genera. Remarkably, the authors present experimental data suggesting that not only bacterial metabolic versatility but also bacterial traits like motility, biofilm formation, iron uptake and the biosynthesis of bioactive secondary metabolites impact bacterial adaptation to a multi‐host lifestyle (Pronk *et al*., 2022). Current hypotheses indicate that beneficial plant‐associated bacteria use this multi‐host evolutionary strategy to ensure survival in highly changing environmental niches (e.g. phyllosphere, rhizosphere).

Current estimates indicate that 75% of leading crops worldwide and 87% of flowering plants depend on animal pollinators for reproduction (Liu *et al*., 2019; Parreño *et al*., 2022). Alarmingly, insect pollinator populations are severely declining as a result of the excessive use of agrochemicals, pathogens, the introduction of invasive species and climate change (Hertel *et al*., 2021; Parreño *et al*., 2022) – an issue that will seriously affect global crop productivity and our capacity to feed the world's growing population. The decline of pollinating insects populations has been also associated with changes in their gut microbiomes (Raymann and Moran, 2018). In this regard, nectar and pollen are a source of microbes for insect pollinators as high number of bacteria exist in floral nectar (up to 10^7^ CFU mm^−3^) and pollen (10^4^–10^7^ CFU g^−1^) (Liu *et al*., 2019). In fact, a significant number of bacterial taxa have been identified as common to honeybee populations and flowers, including different fructophilic lactic bacteria (Liu *et al*., 2019; Crovadore *et al*., 2021) – a recently discovered group of heterofermentative bacteria that prefer fructose over glucose as a carbon source (Filannino *et al*., 2019). Yet, pollen and nectar often contain bioactive plant secondary metabolites (Schmitt *et al*., 2021) as well as agrochemicals that can alter the insect microbiome composition. As a means to understand the role of specific bacterial taxa on honeybee health, a recent report in *Microbial Biotechnology* explored the effects of different agrochemicals and plant secondary metabolites on planktonic and biofilm growth of fructophilic lactobacilli isolated from both the gut of honeybees and bee‐collected pollen (Tlais *et al*., 2022). The study found that the organophosphorus pesticides glyphosate and chlorpyrifos‐methyl completely inhibited planktonic growth of *Apilactobacillus kunkeei* strains, a fructophilic lactobacilli species that is a frequent inhabitant of honeybees, and drastically affected their abilities to form biofilms. On the other hand, neither the insecticide imidacloprid nor the plant secondary metabolites *p*‐coumaric acid and nicotine affected planktonic growth of *A*. *kunkeei*. Alternatively, *p*‐coumaric acid and nicotine had positive effects on the growth of sessile communities. Fructophilic lactobacilli form biofilms in honeybee hosts and previous studies revealed that organophosphorus pesticides have negative impacts on the honeybee microbiome (Motta and Moran, 2020). Tlais *et al*. (2022) found that biofilm‐forming *A*. *kunkeei* strains are more resistant to glyphosate and chlorpyrifos‐methyl, while further studies are required to determine whether these bacteria have beneficial effects on honeybees when exposed to agrochemicals.

Land plants and insects both originated ~475 million years ago (Raven and Wagner, 2021) and the interaction between flowering plants and pollinating insects represents an extraordinary example of co‐evolution. However, what nature has built over millions of years of evolution is in serious threat as a consequence of our human activities. The anthropogenic climate change is seriously affecting global diversity, including plants, insects and their associated microbiota (Parreño *et al*., 2022; Trivedi *et al*., 2022). This partly originates in the continued overuse of chemical pesticides and fertilizers in agriculture, which results in a severe decrease in the diversity of plants and soil microorganisms and the complexity of the interaction networks between plants and microbes (Molina‐Santiago and Matilla, 2020). For this reason, we need urgent solutions to alleviate the negative effects of our human activities, and a greater understanding of plant–microbe interactions will lay the groundwork for the development of novel microorganism‐based approaches aimed at promoting plant productivity and diversity. However, beneficial rhizosphere bacteria share many traits with insect symbionts. Hence, Pronk *et al*. (2022) emphasized that the use of specific rhizosphere bacteria as biopesticides and plant growth promoters should be re‐evaluated since they could potentially disperse to other ecological niches with undesirable effects or have negative impacts on plant‐beneficial insect populations (e.g. pollinators). Consequently, new insights into the ecology and mechanisms that drive the multi‐host lifestyle of beneficial plant‐associated bacteria will likely advance the development of sustainable biotechnological strategies to improve agricultural production.
